# Clinical year veterinary students are concerned about calving cows and request more real‐life, practical exposure to enhance their confidence

**DOI:** 10.1002/vetr.4964

**Published:** 2024-12-26

**Authors:** Jayne Orr, Monika Mihm Carmichael, Rob Kelly

**Affiliations:** ^1^ Scottish Centre for Production Animal Health and Food Safety School of Biodiversity One Health and Veterinary Medicine University of Glasgow Glasgow UK; ^2^ Division of Veterinary Biomedical Sciences Royal Dick School of Veterinary Studies, University of Edinburgh Easter Bush UK

## Abstract

**Background:**

Newly qualified veterinarians are expected to attend emergencies, for example, cow calving, but their clinical exposure to these scenarios before graduation may be limited. This study aimed to investigate what affects veterinary students' confidence and attitudes regarding calving cows.

**Methods:**

Paper questionnaires were distributed to veterinary students in their third (*n* = 240, 2016/2017 and 2017/2018) and fourth years (*n* = 347, 2016/2017, 2017/2018 and 2018/2019) at one UK university to ascertain their demographic data, confidence regarding calving cows (rated on a scale from 1 to 5) and their concerns and suggestions for improving their confidence.

**Results:**

Responses were received from 156 and 300 consenting third‐ and fourth‐year students, respectively. The mean total calving confidence score was higher in the fourth year (34.3/65) than in the third year (30.8/65, *p* < 0.05), and students from both years rarely rated themselves as being ‘confident’ or ‘very confident’ in 13 individual calving tasks. Having some previous experience (odds ratio [OR] 3.34), intending to work with cows (OR 3.25), being from Europe (OR 3) or North America (OR 2.72) and in the fourth year (OR 2.3) increased the odds (*p* < 0.01) of students having some calving confidence. Four main concerns were identified: myself, the task, the animal and the farmer. The students requested more real‐life, practical exposure to improve their confidence.

**Limitations:**

Students were from a single UK veterinary school, and the study relied on self‐reported confidence levels.

**Conclusion:**

Overall, students lacked confidence in calving cows and were concerned about many aspects of this high‐stakes clinical scenario. Veterinary faculty and extramural study providers need to collaborate to develop students' confidence in this area before graduation.

## INTRODUCTION

The Royal College of Veterinary Surgeons (RCVS) and other accrediting bodies state that attending an animal in an emergency is one of the day one competences.^1^, ^2^, ^3^, ^4^ Attending a call to calve a cow is one such emergency for new veterinary graduates, and practitioners have identified this as a skill they would prioritise in veterinary graduates.^5^ Calving a cow is a complex skill combining several steps, from taking a history and carrying out obstetrical manipulations to reviving the calf and dealing with complications.^6^ The complexity of the task combined with a lack of realistic clinical material in an academic setting, variability of student ability within a cohort and the high‐stakes nature of the task mean that teaching this clinical skill can be challenging for veterinary faculty.

Educators also need to recognise the different experiences of students. Veterinary students at entry are predominantly from non‐rural or non‐farming backgrounds,^7^, ^8^ which may affect their prior exposure to calvings. In addition, challenges surrounding ‘seeing practice’ (extramural studies [EMS]), such as the costs/logistics of transport and accommodation and reports of discrimination and harassment on placements, mean that students may not acquire case exposure and clinical experience during their veterinary training.^9^

Confidence is defined in a medical context as a judgement that influences whether an individual is willing (or not) to undertake an activity.^10^ Both being under‐ or overconfident can be detrimental to clinical outcome.^11^ Currently, we do not know how confident veterinary students feel about attending calvings, what influences their confidence and what they think would increase their confidence. Understanding what impacts calving confidence will help inform curriculum development, and identifying the individual steps where students particularly lack confidence will help faculty prioritise those steps in teaching delivery, with the combined benefit of developing both confidence and clinical skills.

Currently, there is a recruitment and retention crisis in the veterinary profession, which is particularly acute in farm animal practice.^12^ Identifying what aspects of calving a cow students look forward to but also are concerned about, or what aspects and where they would like to practise this complex scenario more might inform faculty and EMS providers and encourage students to consider farm animal EMS and a subsequent career in farm animal practice.^12^, ^13^ Therefore, this study first aimed to establish the self‐rated calving confidence levels of veterinary students in their clinical years (specifically the third and fourth years of a 5‐year programme) and any critical demographic variables. It is during this time that veterinary students increasingly pursue clinical EMS (CEMS) opportunities. Second, the study aimed to identify third‐ and fourth‐year students' opinions on the negative (‘concerned about’) and positive (‘look forward to’) aspects of this very practical (but also very high stakes) clinical scenario, as well as the interventions required to enable them to become confident when graduating 1‒2 years later. The approach chosen was a mixed (quantitative and qualitative) questionnaire design.

## MATERIALS AND METHODS

### Context

During the 5‐year veterinary curriculum at the University of Glasgow (UOG), theoretical and practical subjects relevant to animal reproduction and obstetrics in the different species are delivered from years 1 to 4 during production animal and reproduction modules before students enter the mostly clinical and practical final year. Specifically, for farm animal species, students learn the normal reproductive anatomy and physiology with normal parturition lectures and lambing practical classes in the first year and then have three dedicated 1‐hour lectures on calving cows in the third year, followed by scheduled practical classes using a high‐fidelity calving simulator in the clinical reproduction module in the fourth year.

Another compulsory part of their training, and an RCVS requirement, is 12 weeks of preclinical EMS (PCEMS), completed by the end of the second year, and 26 weeks of CEMS, completed by the end of the fifth year (new RCVS EMS policy was implemented in 2024^14^). Students are responsible for arranging their own EMS placements; thus, the species and type of placements undertaken by students are self‐directed. Not all students, therefore, have or seek the opportunity to calve a cow, but all will have undertaken a 2‐week preclinical lambing EMS placement (stipulated by UOG at the time of the study).

### Consent and questionnaire

Students were introduced to this study during a 10‐minute briefing session at the start of their production animal (third year, February 2017 and 2018) and clinical reproduction (fourth year, November 2016, 2017 and 2018) modules, before any teaching was delivered. There were 240 eligible third‐year students overall in 2016 and 2017 and 347 eligible fourth‐year students overall in 2016, 2017 and 2018 (based on numbers of students on class lists). The study was conducted from November 2016 to November 2018; hence, two third‐year cohorts (16/17 and 17/18) and three fourth‐year cohorts (16/17, 17/18 and 18/19) took part. Information on the study was also provided to students via a forum post on their virtual learning environment (Moodle 2). Paper copies of the questionnaire were delivered to students during the briefing session, at which it was stated that participation was entirely voluntary, and completed questionnaires were collected at the end of teaching that day. Fourth‐year students also got the opportunity to complete the questionnaire before they attended a calving practical class. Written consent was obtained within the questionnaire. Students were also asked to enter a personal identifier using the last four digits of their matriculation number and the first letter of their surname, which would allow the linking of questionnaire responses from the third to fourth year without using personal details such as date of birth. For this study, only information on the percentage of fourth‐year students who had also answered the questionnaire in their third year was determined to better understand the composition of the student groups.

The first part of the questionnaire was designed to obtain information on student demographics using a mixture of open and closed questions to determine gender, year of birth, continent of origin, intention regarding the area of veterinary practice they will pursue following graduation (mixed, small, equine, farm animal practice, etc.), previous calving experience and calving confidence. In the ‘previous calving experience’ section, students were asked how many calvings they had (a) observed, (b) assisted with and (c) carried out unassisted. For each, they were given five options: 0, 1‒2, 3‒5, 6‒10 or 10 or more calvings.

In the ‘calving confidence’ section, students were asked to self‐rate their confidence, using a five‐point Likert scale (1—no confidence, 2—little, 3—some, 4—confident, 5—very confident), for 13 individual calving tasks, which included preparation, assessment, manipulation of the calf, extraction of the calf and aftercare of cow/calf, adapted from the description of the steps of calving a cow.^15^ After the first year of the study, to explore student attitudes towards calving further, particularly in relation to enhancing confidence, three free‐text questions were added to the questionnaire from November 2017 (specifically, ‘What do you look forward to?’, ‘What concerns you about calving cows?’ and ‘What would increase your confidence in calving cows?’) and were delivered to the second cohort of third‐year students and the second and third cohorts of fourth‐year students. The questionnaire was pilot tested on 12 final‐year students, and some minor changes were made. A copy of the questionnaire is provided as Supporting Information.

### Data analysis

All entries from paper questionnaires were entered into a Microsoft Excel spreadsheet (version 2405 Build 16.0.17628.20006).

#### Demographic data from the questionnaire

Previous experience was converted into a numerical score, with higher experience scores reflecting students' increased involvement and independence during calving. Specifically, scores were allocated based on the number of calvings students reported (1) they had observed (scores 1‒4), (2) assisted with (scores 5‒8) and (3) carried out unassisted (scores 9‒12). A total experience score was computed for each student by summing up observed, assisted and unassisted scores to a possible maximum of 24. This approach also allowed subsequent categorisation of students for regression analyses, with scores from 5 indicating that the student had at least assisted in calvings and, therefore, had ‘some calving experience’, as opposed to ‘none or minimal experience’ gained only through observations. Similarly, for calving confidence, the Likert scale confidence ratings given to each of the 13 tasks were summed^16^ to compute a total calving confidence (rating) score for each student,^17^ ranging from a minimum of 13 (Likert scale 1—no confidence in all 13 tasks) to a possible maximum of 65 (Likert scale 5—very confident in all 13 tasks; see the questionnaire in Supporting Information). A histogram of all total confidence scores clearly indicated that the data were divided into a total score of less than 27, reflecting a maximum of Likert scale 2—‘little’ calving confidence rating for all tasks, and a score from 27, indicating a Likert scale 3—‘some’ confidence in at least one of the calving tasks. The categorisation of intention, experience and calving confidence for the binary regression analyses is shown in Table 1.

**TABLE 1:** Categorisation of the intention, experience and confidence data gathered from veterinary students in years 3 and 4 of study using a mixture of open and closed questions in a paper‐based questionnaire

Question/parameter	Responses	Categorisation
What is your intention following graduation?	Equine	Would not encounter a cow
	Small animal	
	Wildlife	
	Non‐clinical	
	Do not know	
	Farm animal	Would encounter a cow
	Mixed	
Calving experience total score	0‐4	None or minimal calving experience
	5–24^a^	Some calving experience
How confident do you feel with the following tasks when calving a cow? (Likert scale 1‒5 per task summed to a total score for 13 tasks)	13–26^b^	Little calving confidence
	27–65^c^	Some calving confidence

^a^ Students had assisted in at least 1‒2 calvings or even carried out calvings unassisted and not just observed calvings.

^b^ Students self‐rated an average Likert score of 1 or 2 (having ‘no’ or ‘little’ confidence) in all 13 calving tasks.

^c^ Students self‐rated a Likert score of 3 (having ‘some’ confidence), 4 (‘confident’) or 5 (‘very confident’) in at least one of the 13 calving tasks.

#### Qualitative data

A combination of content and thematic analysis using an inductive explicit, but sometimes also interpretative, approach was performed on responses to the three free‐text questions.^18^, ^19^, ^20^ Data were reviewed manually in Excel by one author initially (J.O.) to establish code descriptors for common words or patterns being observed in the responses. Each code descriptor was given a number and responses were reviewed again to assign this code number; some responses were more comprehensive and thus assigned more than one code number. The responses and code descriptors were then reviewed by a second author (M.M.C.), and a final code description was agreed following an iterative discussion between the two authors. The responses were further reviewed, and responses were re‐coded if needed. Higher‐level themes were then established following discussion and agreement between all three authors. This combined approach allowed the comparison of the frequency of responses assigned to each of the code descriptors between the two year cohorts to identify changes in attitude as students progressed through the clinical years of their programme. A number (#, e.g., 32011) was generated during data entry and given to each free‐text response (e.g., 3—third year, 2—second cohort and 011—the spreadsheet row number); this number identified the student cohort but did not identify the student.

#### Statistical approaches

The analysis was carried out in Minitab (version 19.2020.1). Numerical data were checked for normality and either an ANOVA (for parametric data), Mann‒Whitney test or Kruskal‒Wallis test (for non‐parametric data) was used. Categorical data were analysed using the chi‐squared or Fisher's exact test (if expected counts in the chi‐squared test were less than five). Significance levels were set at a *p*‐value of less than 0.05 unless otherwise stated.

Logistic regression was used to investigate the effects of predictor variables on the outcome of having ‘some calving confidence’. Data from respondents falling into demographic categories with very low numbers were excluded from the univariate and multivariable regression analyses, specifically the ‘would rather not say’ response for gender (*n* = 1) and the ‘Africa’ response for continent of origin (*n* = 9).

Once the final model was determined, manual checks were carried out to establish (1) any confounding effects of age or gender on the variables that remained in the final model, and (2) any plausible interactions between variables in the final model, for example, continent × experience or intention.

## RESULTS

### Student background

**FIGURE 1:**
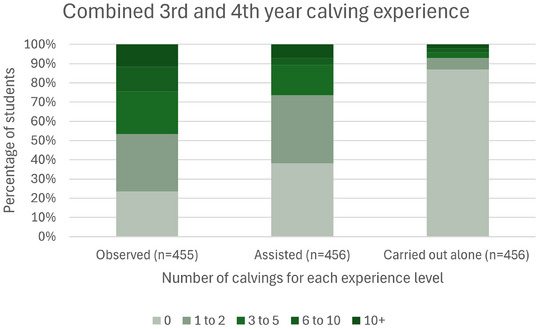
Calving experience levels of third‐ and fourth‐year students combined

Consent to take part in the study was given by 156 (65%) and 300 (86%) third‐ and fourth‐year students, respectively. Forty‐eight percent of consenting fourth‐year students had previously taken part in the study in their third year. A summary of students' demographic characteristics, their intention following graduation and their calving experience scores is shown in Table 2. Apart from students being 1 year older in their fourth year, the distribution of demographic variables (gender, continent and intention following graduation) was similar between third‐ and fourth‐year students. The average calving experience total score was similarly low in both student cohorts (Table 2, *p* > 0.14), with almost 40% of students never having assisted with calving, and almost 90% of students never having carried out a calving unassisted (Figure 1). Only six of the students (1.9%) were clearly very experienced, achieving a maximum experience score of 24.

**TABLE 2:** Comparison of the demographic characteristics of veterinary students taking part in the study in their third and fourth years of the programme

Demographic		Third year, *N* = 156 (34%)	Fourth year, *N* = 300 (66%)	*p*‐Value for difference between third and fourth years
Gender	Male	33 (21%)	67 (23%)	0.759
	Female	123 (79%)	232 (77%)	
	WRNS	Not used	1 (0.3%)	
Age	Mean, 95% CI, ±SD	22.2, 21.75‒22.56, ±2.55	23.4, 23.0‒23.8, ±3.39	<0.001
Continent of origin	Europe	81 (52%)	162 (54%)	0.875
	North America	49 (31%)	96 (32%)	
	Asia	23 (15%)	36 (12%)	
	Africa	3 (2%)	6 (2%)	
Intention following graduation	Would encounter cows	67 (43%)	127 (42%)	0.900
	Would not encounter cows	89 (57%)	173 (58%)	
Calving experience total score (out of a maximum of 24)	Mean, 95% CI, ±SD	5.8, 4.73‒6.82, ±6.62	6.7, 6.00‒7.37, ±6.03	0.142

Abbreviations: CI, confidence interval; SD, standard deviation; WRNS, would rather not say.

### Calving confidence overall and per task

**FIGURE 2:**
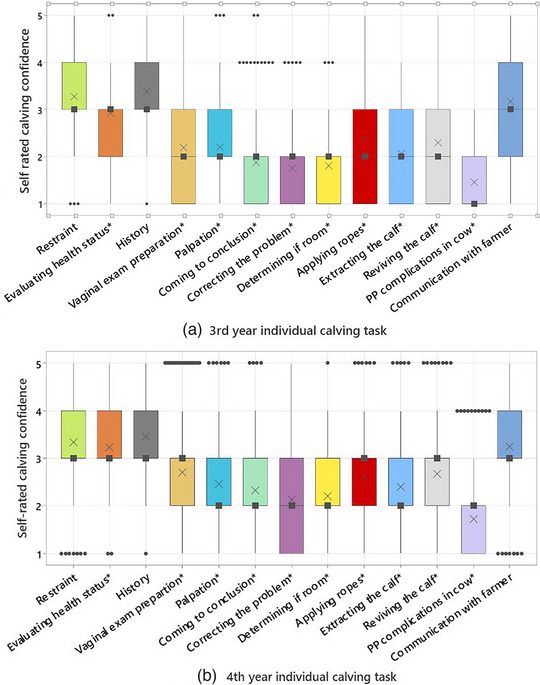
Boxplots of self‐rated confidence of (a) third‐year students and (b) fourth‐year students based on a Likert scale rating (1—no confidence to 5—very confident) for 13 calving‐related tasks. Black squares denote the mean, × denotes the median, black circles denote outliers and * indicates a significant difference (*p* < 0.05) between the third‐ and fourth‐year students in the individual task. Abbreviations: Postpartum (PP)

The total number of responses contributing to this part of the analysis was 435 (146 from third‐year students and 289 from fourth‐year students). Of these students, 99 (23%) were categorised as having ‘little’ calving confidence, and 336 (77%) had ‘some’ calving confidence. The mean total confidence rating score was 30.77 (95% confidence interval [CI] 29.44‒32.11, standard deviation [SD] ±8.39) out of a possible 65 for third‐year students, indicating ‘some’ confidence in at least four to five individual tasks. This score was only slightly higher in fourth‐year students (34.3, 95% CI 33.41‒35.29, SD ±8.23; *p* < 0.05), suggesting a lack of calving confidence overall. None of the individual tasks in either academic year had a mean confidence score above 3.5 out of 5 (Figure 2). Students in both years were least confident in dealing with immediate postpartum complications in the cow, correcting the problem and determining if there is sufficient room to extract the calf. Most tasks showed a slightly higher confidence level (0.2–0.5) in fourth‐year students (*p* < 0.05). The tasks that students were most confident with in both years were history taking, restraint and communication, with no confidence change between the third and fourth years (*p* > 0.05) (see Table S1).

#### What influences calving confidence?

The explanatory variables academic year, gender, continent of origin, career intention following graduation and calving experience passed the criteria for inclusion in the logistic regression model (see Table S2). All variables apart from gender remained in the multivariable model (Table 3). Having some experience and intending to work with cows, as well as being from Europe or North America and in the fourth year of the programme all independently increased the odds of having ‘some’ calving confidence two‐ to threefold (Table 3). Neither age nor gender exerted any confounding effects on the variables in the final model. When plausible interactions between continent of origin, experience and intention were investigated in the final model, neither age nor gender enhanced the odds of students being categorised as having ‘some’ confidence beyond the sum of the odds ratios for the individual variables.

**TABLE 3:** Multiple logistic regression for variables associated with having ‘some' or more calving confidence

Variable	Odds ratio	95% confidence interval	*p*‐Value
Accademic year			
Third (*n* = 146)	Reference		0.002
Fourth (*n* = 289)	2.30	1.37–3.87	
Continent of origin			
Asia (*n* = 59)	Reference		
Europe (*n* = 234)	3.00	1.49–6.05	0.002
North America (*n* = 142)	2.72	1.36–5.44	0.005
Intention following graduation			
Would not encounter cows (*n* = 185)	Reference		<0.001
Would encounter cows (*n* = 250)	3.25	1.75–6.05	
Calving experience^a^			
None/minimal (*n* = 163)	Reference		<0.001
Some (*n* = 271)	3.34	1.94–5.77	

^a^ No data for one student.

### Qualitative data analysis

Fifty‐one out of 52 consenting third‐year students (2017) and 111 of 114 (2017) and 96 of 99 (2018) consenting fourth‐year students provided free‐text responses.

#### Concerns about calving

**FIGURE 3:**
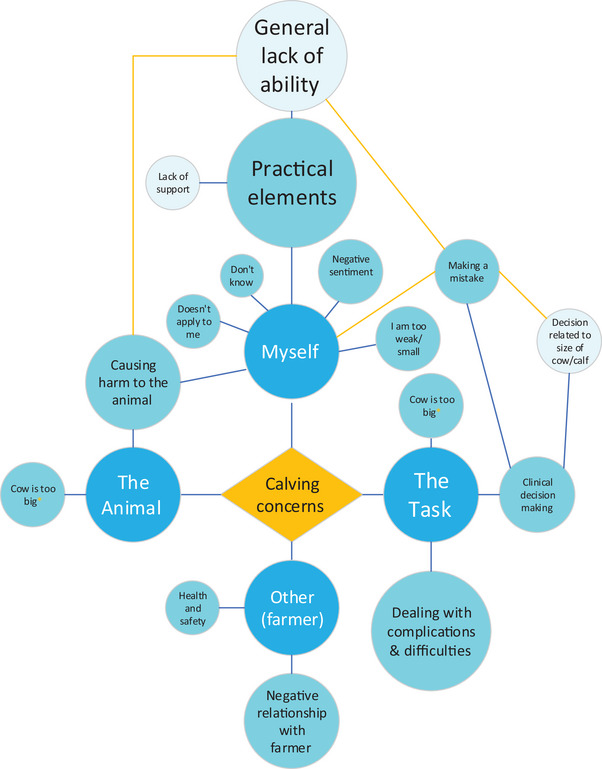
Coding diagram showing the main themes (dark blue circles) and subthemes (light blue circles) that emerged when students were asked ‘What aspects of calving a cow concern you as a new graduate’? The size of each circle approximates the frequency of response. Orange lines and denote interlinking themes/subthemes

The responses related to the (often very personal) stresses of encountering a difficult situation that the student did not (yet) feel trained for (‘being concerned about everything’), as well as possible negative consequences for the animals and the farmer, all of which could be encoded by 11 sometimes interlinked code descriptors under four main themes: myself, the task itself, the animal and other (the farmer) (Figure 3 and Table S3). Students made mostly general statements about dealing with complications or their general lack of ability (‘mistakes’, ‘difficult’ and ‘can't do’), but also provided detail clearly showing that they had attended calvings or remembered prior teaching content (‘calf size’ or ‘C‐section’ decisions, ‘experienced farmer’).

The third‐year cohort mentioned concerns about clinical decision making more than fourth‐year students, while fourth‐year students mentioned causing harm to the animal and a negative relationship with the farmer more than third‐year students (Table S3). Only a few students stated concerns about ‘not having back up’ and ‘health and safety’ on‐farm.

‘Understanding what's going wrong/where to start. Understanding the farmers capabilities and expectations and being underestimated’ (third year, # 32011).

‘Not knowing what to do. Intimidation from (experienced) farmers with little patience’ (fourth year, # 43092).

#### What aspects of calving a cow do students look forward to as a new graduate?

**FIGURE 4:**
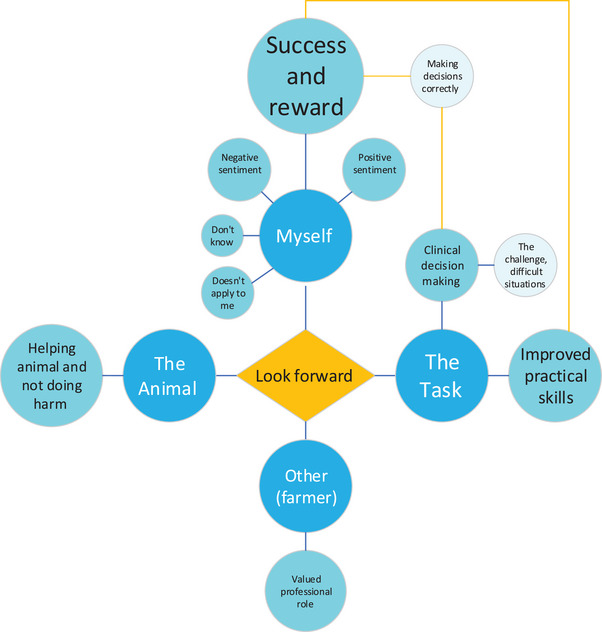
Coding diagram showing the main themes (dark blue circles) and subthemes (light blue circles) that emerged when students were asked ‘What aspects of calving a cow do you look forward to as a new graduate’? The size of each circle approximates the frequency of response. Orange lines denote interlinking themes/subthemes

Interestingly, responses indicated that concerns stated in the previous question could, if addressed, turn around and become very worthwhile experiences. Hence, the same four themes summarised all responses with only nine sometimes interlinked code descriptors (Figure 4 and Table S4). Student‐ and animal‐related positive sentiment represented the majority of the responses (being rewarded/experiencing success, being able to improve animal welfare), but there was also mention of skill and technical calving aspects.

Third‐year students mentioned clinical decision making and associated solutions more than fourth‐year students, while fourth‐year students mentioned practical elements more than third‐year students (see Table S4).

‘I think it will be exciting figuring out how to get the calf out. Once the calf is out, there is a sense of accomplishment’ (third‐year student, # 32031).

‘The reward of successful delivery and a healthy calf and having a happy healthy cow. Also, the high challenge/reward of determining what's wrong and correcting it’ (fourth‐year student, # 43032).

#### What would increase student calving confidence?

**FIGURE 5:**
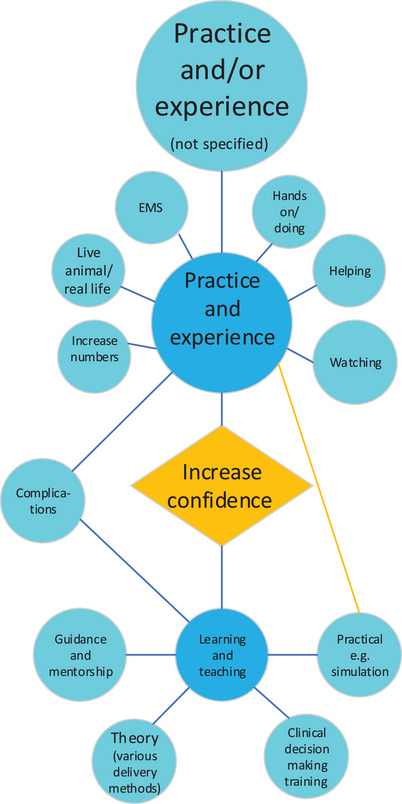
Coding diagram showing the main themes (dark blue circles) and subthemes (light blue circles) that emerged when students were asked ‘What do you think would increase your confidence in calving cows’? The size of the circle approximates the frequency of response. The orange line denotes interlinking themes/subthemes

Two main themes related to (1) practice and experience or (2) intramural teaching and learning (in a university environment) summarised responses coded into 10 subthemes or code descriptors (Figure 5 and Table S5). The overwhelming student response was that ‘more practice’ and/or ‘more experience’doing or learning to do a calving would increase their confidence. In relation to what they wanted to gain from each theme, responses ranged from very general statements (‘practice’, ‘experience’, ‘real’, easy to difficult scenarios, ‘lots’) to the provision of a little more detail (mention of specific skills, equipment or presentations, complex scenarios and cow clinical signs). However, responses were very detailed regarding how they wanted to ‘practice/gain experience’ (related to learning environment, degree of involvement, independence and guidance) and ‘learn or be taught’ (related to theory, curriculum design, rotations, sensory experience or actual skills) (Figure 5 and Table S5).

Students also referred to the degree of physical involvement (‘observe’ and ‘hands on’), supervision (‘under guidance’ and ‘on my own’) and clinical involvement (‘I am making decisions’). In fact, students mentioned using a simulator for practical training, and one response even listed an immersive experience together with communication skills. Students also specified that they wanted to obtain more theoretical knowledge with videos, lectures or other resources (third‐year students, in particular).

‘Hearing lectures, watching videos and having a practical on calving’ (third‐year student, # 32012).

‘More experience with calving cows. Guidance on how to do it properly and how to rectify my problems’ (fourth‐year student, # 43049).

See Table S5 for more detailed qualitative analysis results.

## DISCUSSION

This study demonstrated that over 40% of veterinary students, while in the clinical phase (years 3 and 4) of their 5‐year programme, intend to work with cows following graduation. Despite this interest in farm animal work among a large proportion of veterinary students during the period of the study, student experience in calving cows was low overall, with almost a quarter of third‐ and fourth‐year students not even observing a calving. No prior data exist on the calving experience of veterinary students. However, student experience in the current study is similar to that reported in year 4 and 5 students in a Swedish veterinary programme, where 68% had assisted with a feline ovariohysterectomy, similar to the 67% of fourth‐year students in the current study who had assisted in a calving.^17^ A relatively recent study evaluating bovine rectal examination experience showed that 65% of fourth‐year students had no experience, but a direct comparison is difficult as rectal examination is not a task where students can easily assist veterinarians or farmers side by side.^21^

Overall calving confidence levels were low (with minimal differences between third‐ and fourth‐year students); however, the study identified that coming from Europe or North America, having some calving experience and intending to work with cows positively influenced students' calving confidence. Interactions between experience, continent and intention were found, with experienced students tending to want to work with cows and coming from North America or Europe. Further research would be needed to unpick what drives this relationship between the three variables. It could even be argued that a lack of confidence does not matter if the student is not interested in a career that would involve farm animals; however, the approach to this clinical task is transferable to several other species and clinical skills, and it is thus important to determine whether demographic or experience variables most influence student learning and confidence.

All students at UOG (at the time this study was conducted) had to undertake a 2‐week lambing PCEMS placement, allowing transfer of lambing skills to calving skills with students having the option to partake in CEMS where exposure to calving cows might also be obtained. The intention to work with cows could drive students to show an interest in obstetrics and obtain EMS placements where calving cows will be encountered. Furthermore, students brought up on a farm likely feel better equipped to deal with such an emergency. Seeing that even the lowest total confidence (rating) scores indicated ‘little’ or ‘some’ confidence in one or more tasks in our study despite a complete lack of real‐life calving experience, we can assume that students will acquire ‘little’ or ‘some’ confidence from prior lectures, practical classes and self‐directed learning provided by the curriculum alone. In this context, any influences of EMS or farming background would provide an interesting additional insight into determinants of calving confidence in veterinary students, but these were not investigated here. Interestingly, identifying as male tended to increase students' odds of having some or more calving confidence, but gender was not found to influence calving confidence in the multivariant analysis. Previously, females were found to feel less able to pursue a career in farm animal practice, despite the veterinary profession increasingly being a predominately female workforce.^22^

There were variations in confidence levels for the individual tasks associated with calving cows, with some of the tasks the students had lower confidence in also identified as subthemes within the thematic analysis of student concerns, for example, dealing with postpartum complications in the cow. Clearly, while some of the calving tasks lend themselves to being taught within the curriculum using a high‐fidelity calving simulator, for example, applying calving ropes, other tasks are more difficult to simulate, for example, dealing with the complication ‘bleeding’, which is specifically mentioned by some students. These types of complex and high‐stakes tasks would need to be encountered on EMS and would be reliant on the student ‘being in the right place at the right time’. This links with the overriding response from especially fourth‐year students asking for more practice/experience or ‘real‐life’ exposure and higher case numbers to increase their confidence. Clearly, fourth‐year students are starting to see the bigger picture and recognise that they could be called to calve a cow in approximately 18 months. While exposure to ‘real life’ might be seen as valuable by students in terms of building confidence, it can be problematic. A calving is a stressful event for everyone involved, with the lives of the cow and calf at stake (and arguably the lives of the veterinarian and farmer if the situation is unsafe). Calls to calve cows often happen out of hours when the EMS student is no longer at the practice, and if the student is there, the farmer and/or veterinarian might be reluctant for the student to ‘have a go’. However, if students are equipped with knowledge and skills from didactic calving teaching, video demonstrations of calving presentations and practical skill training using high‐fidelity models, then they may feel more confident at stepping forward during a calving on EMS provided that they are guided by a supportive mentor.

A positive experience calving a cow on an EMS placement when accompanied by such a mentor can make all the difference for students in deciding whether they should pursue a career in farm animal or mixed practice.^23^, ^24^ Many of the themes identified in this study (albeit only investigating one specific farm skill), had parallels with the results of thematic analysis of 1146 responses from the UK and Irish veterinary schools investigating why students feel able (or unable) to pursue a career in farm animal practice.^22^ Given the current recruitment and retention issues facing the industry, we need to encourage young veterinarians into the profession.^12^, ^13^ In the fourth year in particular, students recognised the veterinarian‒farmer relationship as a concern, which relates to veterinarians reporting being perceived as ‘doing a good job’ by the farmer, thereby building the key trust relationship between veterinarians and farmers, as one challenge of being a farm animal veterinarian.^25^ The reasons cited by veterinarians for leaving farm animal practice include the feeling that they are ‘not good enough’ and a ‘lack of confidence in dealing with emergencies’, based on an online survey of farm animal veterinarians who both stayed in and left the profession.^26^ It was reassuring that despite all the concerns voiced, when students were asked what they look forward to when calving a cow, they recognised some of the positive aspects, such as the feeling of success/reward, improving animal welfare and improving their practical skills. These positive aspects should be nurtured and used to encourage veterinary students into a career in farm animal practice.

The strengths of this study were the high response rate to the questionnaire and the collection of data over more than one academic year (although qualitative data were collected from only one cohort of third‐year students). The tasks used for self‐rated calving confidence were based on a published list of tasks associated with calving created using cognitive task analysis of expert bovine practitioners.^15^

Measuring confidence by asking students to report how confident they feel using a paper questionnaire with limited descriptors for each level may not be a true reflection of a student's actual confidence or capabilities when they are faced with a real‐life calving, as some may inherently be over‐ or underconfident and misjudge themselves. Furthermore, knowing what weighting should be applied to each individual calving task and what the difference between levels of confidence equates to in terms of the more complex concept of capability remains unknown.^27^ The nature of thematic analysis, combined with the demographics of two of the researchers performing the analysis (white, female) and their relationship with the participants (lecturers), may also add some unintentional bias to the analysis of the qualitative data. Future studies could investigate additional demographics, such as ethnic group and urban versus rural background, as well as the impact of the number of weeks and types of EMS carried out, seeing that real‐life practice was mentioned so often by students in terms of increasing their calving confidence.

While the participants of this study were veterinary students, employers of new and recent graduates could also use the results to inform their further graduate development. There is a role for university‐delivered calving skills training with high‐fidelity simulation and immersive scenarios prior to the final year, with the advantage of simulator training guaranteeing consistency and not requiring live animals when training large student cohorts. Clinical students want real‐life exposure, but they also recognise that the scenarios are complex, with animals’ welfare and the veterinarian‒farmer relationship at stake, and their biggest concern is to ‘fail’ and misjudge and mismanage. However, only (increasingly involved and supervised) exposure to real cases will capture the complexity of clinical judgement required during cow calving and thus elevate students' confidence in their final year as an essential prerequisite for entering farm animal practice. We propose stronger links between farm animal practitioners at university and in practice to address the challenge of students ‘being confident at calving a cow’ at the end of their clinical training.

## AUTHOR CONTRIBUTIONS

Jayne Orr was responsible for conception, design, acquisition of data, analysis, interpretation, drafting of the manuscript and final approval. Monika Mihm Carmichael was responsible for conception, design, analysis, interpretation, critical revision of the manuscript and final approval. Rob Kelly was responsible for conception, design, interpretation, critical revision of the manuscript and final approval.

## CONFLICT OF INTEREST STATEMENT

The authors are not aware of any conflicts of interest with this work.

## FUNDING INFORMATION

The authors received no specific funding for this work.

## ETHICS STATEMENT

Ethical approval was granted from the UOG Ethics Committee (application number 20016009), which also stipulates adherence to UK data protection legislation regulating secure storage and access to research data.

## Supporting information



Supporting Information

Supporting Information

Supporting Information

Supporting Information

Supporting Information

Supporting Information

## Data Availability

The data are available upon request from the authors.
