# Supplementary material for: Clinical year veterinary students are concerned about calving cows and request more real‐life, practical exposure to enhance their confidence
Source: Vet Rec. 2024 Dec 26;196(11):e4964. doi: 10.1002/vetr.4964 (PMC12124102; doi:10.1002/vetr.4964)
Supplement: Supplementary file 1 — Supporting Information [file VETR-196-e4964-s004.docx]

Supplementary Table 1 – Comparison of self-rated calving confidence between 3^rd^ and 4^th^ year students. Confidence Interval (CI), Standard Deviation (SD).

| Calving task | 3^rd^ year confidence  Mean  95% CI  +/- SD | 4^th^ year confidence  Mean  95% CI  +/- SD | Mann Whitney  p value |
| --- | --- | --- | --- |
| Restraint | 3.3  3.1 – 3.4  +/- 0.98 | 3.3  3.2 – 3.4  +/- 0.88 | 0.490 |
| Establishing health status | 2.9  2.8 – 3.0  +/- 0.72 | 3.2  3.1 – 3.3  +/- 0.67 | 0.000 |
| History taking | 3.4  3.3 – 3.5  +/- 0.71 | 3.5  3.4 – 3.5  +/- 0.77 | 0.415 |
| Vaginal exam | 2.2  2.0 – 2.4  +/- 0.99 | 2.7  2.6 – 2.8  +/- 1.11 | 0.000 |
| Palpation | 2.2  2.0 – 2.3  +/- 0.95 | 2.5  2.3 – 2.6  +/- 1.04 | 0.014 |
| Coming to a conclusion | 1.9  1.7 – 2.0  +/- 0.97 | 2.3  2.2 – 2.4  +/- 0.91 | 0.000 |
| Correcting the problem | 1.7  1.6 – 1.9  +/- 0.83 | 2.1  2.0 – 2.2  +/- 0.88 | 0.000 |
| Assessing for room | 1.8  1.7 – 1.9  +/- 0.81 | 2.2  2.1 – 2.3  +/- 0.87 | 0.000 |
| Applying ropes | 2.4  2.2 – 2.5  +/- 1.12 | 2.6  2.5 – 2.7  +/- 0.99 | 0.022 |
| Extracting the calf | 2.1  1.9 – 2.2  +/- 1.01 | 2.4  2.3 – 2.5  +/- 0.96 | 0.001 |
| Reviving the calf | 2.3  2.1 – 2.5  +/- 1.14 | 2.7  2.6 – 2.8  +/- 1.00 | 0.000 |
| Dealing with post-partum complications | 1.5  1.4 – 1.6  +/- 0.65 | 1.7  1.6 – 1.8  +/- 0.78 | 0.001 |
| Communication with the farmer | 3.2  3.0 – 3.3  +/- 1.01 | 3.2  3.1 – 3.3  +/- 0.89 | 0.630 |
