# Supplementary material for: Clinical year veterinary students are concerned about calving cows and request more real‐life, practical exposure to enhance their confidence
Source: Vet Rec. 2024 Dec 26;196(11):e4964. doi: 10.1002/vetr.4964 (PMC12124102; doi:10.1002/vetr.4964)
Supplement: Supplementary file 2 — Supporting Information [file VETR-196-e4964-s003.docx]

Supplementary Table 2 Calving confidence univariate analysis. Confidence Interval (CI), Standard Deviation (SD).

| Variable | | n = 435 | | | Odds Ratio  (95% CI) | P value |
| --- | --- | --- | --- | --- | --- | --- |
|  |  | Little  Confidence | Some or more confidence | Total |  |  |
|  |  | 99 | 336 | 435 |  |  |
| Study Year | 2016/17 | 48 (27%) | 132 (73%) | 180 | Reference | - |
|  | 2017/18 | 34 (21%) | 126 (79%) | 160 | 1.35  (0.82 – 2.23) | 0.245 |
|  | 2018/19 | 17 (18%) | 78 (82%) | 95 | 1.67  (0.90 – 3.10) | 0.106 |
| Academic year | 3^rd^ | 49 (34%) | 97 (66%) | 146 | Reference | - |
|  | 4^th^ | 50 (17%) | 239 (83%) | 289 | 2.41  (1.53 – 8.82) | 0.000 |
| Age | Mean ^a^  (95% CI, +/- SD) | 23.1  22.6 – 23.7  +/- 2.76 | 22.9  22.5 – 23.2  +/- 3.27 | NA | 0.97  (0.91 – 1.04) | 0.460 |
| Gender | Female | 84 (25%) | 257 (75%) | 341 | Reference | - |
|  | Male | 15 (16%) | 79 (84%) | 94 | 1.72  (0.94 – 3.15) | 0.078 |
| Continent | Asia | 30 (51%) | 29 (49%) | 59 | Reference | - |
|  | Europe | 34 (15%) | 200 (85%) | 234 | 6.09  (3.25 – 11.39) | 0.000 |
|  | North America | 35 (25%) | 107 (75%) | 142 | 3.16  (1.67 – 5.99) | 0.000 |
| Intention | No cows | 17 (9%) | 168 (91%) | 185 | Reference | - |
|  | Cows | 82 (33%) | 168 (67%) | 250 | 4.82  (2.74 – 8.48) | 0.000 |
| Experience | None/  minimal | 69 (42%) | 94 (58%) | 163 | Reference | - |
|  | Something | 30 (11%) | 241 (89%) | 271 | 5.90  (3.61 – 9.63) | 0.000 |
