# Supplementary material for: Clinical year veterinary students are concerned about calving cows and request more real‐life, practical exposure to enhance their confidence
Source: Vet Rec. 2024 Dec 26;196(11):e4964. doi: 10.1002/vetr.4964 (PMC12124102; doi:10.1002/vetr.4964)
Supplement: Supplementary file 3 — Supporting Information [file VETR-196-e4964-s001.docx]

Supplementary Table 3 – Code number, descriptions, frequency of code being identified within 3^rd^ and 4^th^ year student responses and subsequent theme development for the question - What aspects of calving a cow concern you as a new graduate?

| Code no | 3^rd^ year Number of times code mentioned within responses | % | 4^th^ year Number of times code mentioned within responses | % | Code Description/sub theme | Theme |
| --- | --- | --- | --- | --- | --- | --- |
| 1 | 11 | 15 | 74 | 23 | Lack of general clinical (including skill deficit) ability | MYSELF |
| 2 | 5 | 7 | 15 | 5 | Physically too demanding | TASK and MYSELF |
| 3 | 12 | 17 | 62 | 19 | Encountering complicated clinical scenario, general and very specific, incl management | TASK |
| 4 | 8 | 11 | 39 | 12 | (Causing) detriment(al) or risk to animal welfare/life, specifics and general sentiment (‘when it goes wrong’) | ANIMAL |
| 5 | 12 | 17 | 28 | 9 | (Mistakes in) clinical decision making (incl caesarean – but in general, not ‘pull or calve versus c section’), consequences of decision making = responsibility, lack of knowledge | TASK and MYSELF |
| 6 | 5 | 7 | 26 | 8 | (Mistakes in) calf size decision making incl caesarean (‘pull or do c section’) | TASK and MYSELF |
| 7 | 9 | 13 | 38 | 12 | Vet-Farmer relationship (breakdown), negative vet-farmer scenario | OTHER (farmer) |
| 8 | 1 | 1 | 4 | 1 | Health and safety concerns | OTHER (H&S) |
| 9 | 3 | 4 | 12 | 4 | Everything – negative emotional state and stress | MYSELF |
| 10 | 2 | 3 | 4 | 1 | Not applicable | MYSELF |
| 11 | 2 | 3 | 1 | 0.3 | Undecided | MYSELF |
| 0 | 2 | 3 | 15 | 5 | No data | No response |
| Total | 72 |  | 318 |  |  |  |
