# Supplementary material for: Clinical year veterinary students are concerned about calving cows and request more real‐life, practical exposure to enhance their confidence
Source: Vet Rec. 2024 Dec 26;196(11):e4964. doi: 10.1002/vetr.4964 (PMC12124102; doi:10.1002/vetr.4964)
Supplement: Supplementary file 4 — Supporting Information [file VETR-196-e4964-s005.docx]

Supplementary Table 4 Code number, descriptions, frequency of code being identified within 3^rd^ and 4^th^ year student responses and subsequent theme development for the question - What aspects of calving a cow do you look forward to as a new graduate?

| Code  no | 3^rd^ year Number of times code mentioned within responses | % | 4th year Number of times code mentioned within responses | % | Code Description/  sub theme | Theme |
| --- | --- | --- | --- | --- | --- | --- |
| 1 | 9 | 11 | 51 | 16 | Practical skill improvement | TASK |
| 2 | 22 | 28 | 101 | 32 | Personal fulfilment and professional Reward - Success | MYSELF |
| 3 | 8 | 10 | 23 | 7 | Valued Professional role | OTHER |
| 4 | 10 | 13 | 36 | 11 | Clinical decision making and solutions | TASK and MYSELF |
| 5 | 16 | 20 | 56 | 18 | Animal welfare | ANIMAL and MYSELF |
| 6 | 2 | 3 | 2 | 1 | Undecided | MYSELF |
| 7 | 1 | 1 | 6 | 2 | Nothing  (negative sentiment) | MYSELF |
| 8 | 1 | 1 | 5 | 2 | Everything  (positive sentiment) | MYSELF |
| 9 | 4 | 5 | 5 | 2 | Not applicable to me | MYSELF |
| 0 | 7 | 9 | 34 | 11 | No data | No response |
| Total | 80 |  | 319 |  |  |  |
