# Supplementary material for: Clinical year veterinary students are concerned about calving cows and request more real‐life, practical exposure to enhance their confidence
Source: Vet Rec. 2024 Dec 26;196(11):e4964. doi: 10.1002/vetr.4964 (PMC12124102; doi:10.1002/vetr.4964)
Supplement: Supplementary file 5 — Supporting Information [file VETR-196-e4964-s006.docx]

Supplementary Table 5 Code number, descriptions, frequency of code being identified within 3^rd^ and 4^th^ year student responses and subsequent theme development for the question - What do you think would increase your confidence in calving cows? Not applicable (NA)

| Code no | Total response 3^rd^ year | % | Total response 4^th^ year | % | Code description/sub theme | Theme |
| --- | --- | --- | --- | --- | --- | --- |
| 1 | 33 | 59 | 132 | 45 | Practice/experience (non-specific) | Practice/experience |
| 1a | 4 | 5 | 25 | 9 | Practice/experience - Hands on/doing | Practice/experience |
| 2 | 4 | 5 | 15 | 5 | Practice/experience - Live animal or real life element | Practice/experience |
| 2a | 3 | 4 | 19 | 6 | Practice/experience - EMS | Practice/experience |
| 3 | 8 | 11 | 29 | 10 | Further teaching – simulation or practical | Teaching and learning |
| 4 | 1 | 1 | 12 | 4 | Increased case load/seeing/doing ‘lots’ more | Practice/experience |
| 5 | 1 | 1 | 9 | 3 | helping (more) | Practice/experience |
| 6 | 4 | 5 | 12 | 4 | watching (more) | Practice/experience |
| 7 | 3 | 4 | 16 | 5 | Feedback, low stress, support and guidance (can be specific) while doing | Teaching and learning |
| 8 | 11 | 15 | 13 | 4 | Further teaching - theoretical knowledge, resources, assessment, general | Teaching and learning |
| 9 | 0 | 0 | 0 | 0 | Code no longer used | NA |
| 10 | 3 | 4 | 10 | 3 | Complication management, decision making | Practice/experience and teaching and learning |
| Total | 75 |  | 293 |  |  |  |
