# Supplementary material for: Clinical year veterinary students are concerned about calving cows and request more real‐life, practical exposure to enhance their confidence
Source: Vet Rec. 2024 Dec 26;196(11):e4964. doi: 10.1002/vetr.4964 (PMC12124102; doi:10.1002/vetr.4964)
Supplement: Supplementary file 6 — Supporting Information [file VETR-196-e4964-s002.pdf]

Evaluation of calving simulator training in the veterinary undergraduate curriculum as part of a blended learning programme

Consent

I give consent for my anonymized data to be used and understand that a unique identification number will only have to be used to match before and after questionnaires.

I understand that my skills may be assessed in a formative OSCE (BVMS4) or by the practice vet (BVMS5).

I understand I can withdraw at any time.

I have read the information sheet/attended information lecture and have taken the opportunity to ask any questions if necessary.

☐ Tick box

Questionnaire

A) Background Information

1. ID number________________________________
   (Last 4 digits of Matriculation number and first initial of surname)

2. Gender (please circle)

   Male Female Other Would rather not say

3. Year of birth___________________________________

4. Continent of origin (please circle)

   Asia Australasia Africa Europe North America South America

5. What is your intention following graduation? (please circle)

   Small Equine Farm Mixed Non-clinical Don't know

B) Confidence level self-assessment

6. How confident do you feel with the following tasks (a – m) when calving a cow? (Please tick one box for each of the listed tasks)

	Confidence level				
**Tasks**	**No confidence**	**Little Confidence**	**Some confidence**	**Confident**	**Very confident**
	**1** (not confident)	**2**	**3**	**4**	**5** (very confident)
a) Restraint of the cow					
b) Evaluating the cow's current health status					
c) History taking					
d) Preparing the cow for vaginal examination					
e) Palpation of vagina/cervix/fetus					
f) Coming to a conclusion about the obstetrical problem					
g) Correcting the obstetrical problem					
h) Determining if sufficient room to extract the calf					
i) Applying the head rope, leg ropes and calving aid					
j) Extracting the calf					
k) Reviving the calf					
l) Dealing with immediate postpartum complications in the cow (eg. bleeding)					
m) Communicating with the farmer					

C) Previous Experience:

7. How many calvings needing assistance have you **observed** (Didn't get to do anything practical)? (please circle)

   0 1-2 3-5 6-10 10+

8. How many calvings needing assistance have you **helped** with (with some direction from vet/teacher/farmer)? (please circle)

   0 1-2 3-5 6-10 10+

9. How many calvings needing assistance have you **carried out with no help** (i.e. no direction from vet/teacher/farmer)? (please circle)

   0 1-2 3-5 6-10 10+

D) Student Opinion

10. What aspects of calving a cow do you **look forward to** as a new graduate?

11. What aspects of calving a cow **concern you** as a new graduate?

12. What do you think would increase your **confidence** in calving cows?

13. What do you think would increase your **technical ability** in calving cows?

14. Any other comments?
